# Are you ready for the tick season? Spring dynamic of tick diversity and density in urban and suburban areas

**DOI:** 10.1186/s13071-025-06793-0

**Published:** 2025-04-19

**Authors:** Dagmara Wężyk, Dorota Dwużnik-Szarek, Maciej Kowalec, Szymon Biela, Kajetan Biernacki, Adrian Macion, Zofia Mencwel, Natalia Safarzynska, Liliana Sajnok, Weronika Słomka, Anna Bajer

**Affiliations:** 1 Department of Eco-Epidemiology of Parasitic Diseases, Institute of Developmental Biology and Biomedical Sciences, Faculty of Biology, University of Warsaw, Miecznikowa 1, 02-096 Warsaw, Poland; 2 Department of Bacterial Genetics, Institute of Microbiology, Faculty of Biology, University of Warsaw, Miecznikowa 1, 02-096 Warsaw, Poland

**Keywords:** *Dermacentor*, *Ixodes*, Spring dynamic, Tick density, *Borrelia*, *Rickettsia*, City, Warsaw, Poland

## Abstract

**Background:**

Occurrence of tick-borne diseases (TBD) is often seasonal and associated with seasonal activity of appropriate tick vectors. As seasonal activity of ticks differs, the risk of contracting particular TBD should change between and within seasons. It is of key importance to monitor seasonal dynamic of tick vectors, especially in human-associated habitats. The aim of the current study was to compare activity and density of *Ixodes ricinus* and *Dermacentor reticulatus* during spring season in urban and suburban habitats.

**Methods:**

Systematic tick collection by dragging was performed every 1–2 weeks between mid-March and mid-June 2021 at 15 sites: 6 in Warsaw (urban areas) and 9 in suburban areas.

**Results:**

During 178 field collections of ticks, including 131 collections from urban sites and 47 collections from rural areas, 738 ticks (385 adult *D. reticulatus* and 353 *I. ricinus*) were collected. *Dermacentor reticulatus* ticks are found from the beginning of spring, peaking in April and May, and *I. ricinus* ticks are present from early April, peaking in April and May as well. *I. ricinus* were abundant in rural and urban areas, including botanical garden and forest kindergarten area. *Dermacentor reticulatus* were found in urban fallow lands but were not collected in parks. These ticks were abundant in fallow lands, meadow, and mixed forest. DNA of *B. burgdorferi* s.l. and *Rickettsia* spp. was identified in ticks from urban areas.

**Conclusions:**

Due to the marked differences in spring dynamic of *D. reticulatus* and *I. ricinus*, the sampling effort should be repeated at least three times per season for accurate estimation of tick occurrence (presence/absence) and density. Due to “exchange” of tick species, total tick density remains high through the spring season of activity, which may result in high transmission of tick-borne pathogens (TBPs). Tick densities are dependent on the habitat type and may be low in well-managed agricultural habitats (crop fields, pastures, chicken yard), but high in semi-natural habitats (fallow lands, rural forests). Numerous *I. ricinus* populations can be maintained in urban green areas such as botanical gardens. Ticks from urban areas can serve as vectors of important TBPs (*B. burgdorferi* s.l., *Rickettsia* spp.).

**Graphical Abstract:**

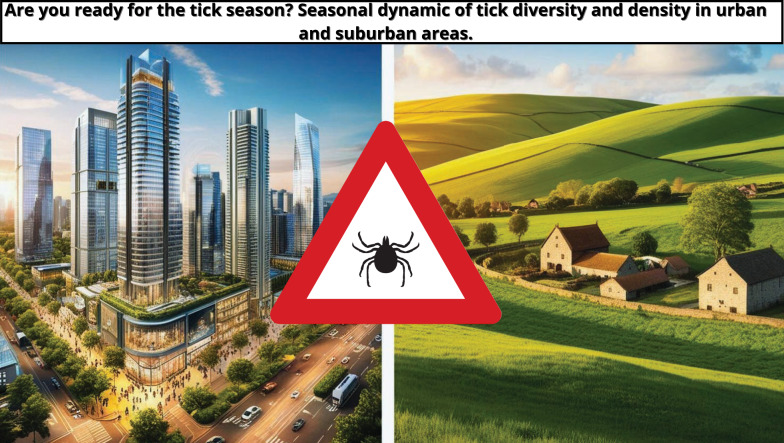

**Supplementary Information:**

The online version contains supplementary material available at 10.1186/s13071-025-06793-0.

## Background

Ticks and tick-borne diseases, i.e., Lyme disease or babesiosis, constitute the growing problem in the Northern Hemisphere, including Europe, affecting humans and animals [1, 2]. For example, the European Parliament, in its resolution of 15 November 2018 on Lyme disease/borreliosis, recognized the disease as an alarming European health problem; a silent epidemic with around 1 million citizens suffering from the disease, according to the census method (https://eur-lex.europa.eu/legal-content/EN/TXT/PDF/?uri=CELEX:52018IP0465&rid=3). Babesiosis is an emerging infectious disease, affecting humans, livestock, and pets [2–4]. Other tick-borne bacteria (*Rickettsia* spp.) and viruses (TBEV) are also of public health concerns [5, 6].

Occurrence of TBD is often seasonal and associated with seasonal activity of appropriate tick vectors [7, 8]. As seasonal activity of ticks differs, the risk of contracting particular TBD should change between and within seasons and it is of key importance to monitor seasonal dynamic of tick vectors, especially in human-associated habitats.

In Poland, two species of ticks play the most important role as vectors of TBDs: the common tick, *Ixodes ricinus*, and the ornate dog tick, *Dermacentor reticulatus* [9]. These two species differ in preferred habitats, seasonal activity, and range of vectored pathogens [10, 11]. *Ixodes ricinus* plays a major role in transmission of borreliae, TBEV, and three species of zoonotic babesiae [11–13], while *D. reticulatus* is the major vector of *Babesia canis* and *Rickettsia raoulti* [14, 15].

There are not many studies comparing seasonal activity of these two tick species, especially regarding relative changes in tick densities within the spring season, which is the main season of tick activity, following winter diapause. Our study was planned to monitor changes in tick density within the spring season in 2021. The focus of the current study was on comparison of tick activity in human-associated habitats in the capital city of Warsaw and in its suburban areas. In suburban areas the effect of different management methods was also determined by comparison of tick densities in different types of agricultural lands. Finally, the seasonal dynamic of males and females within tick species was also monitored and compared.

## Methods

**Fig. 1:**
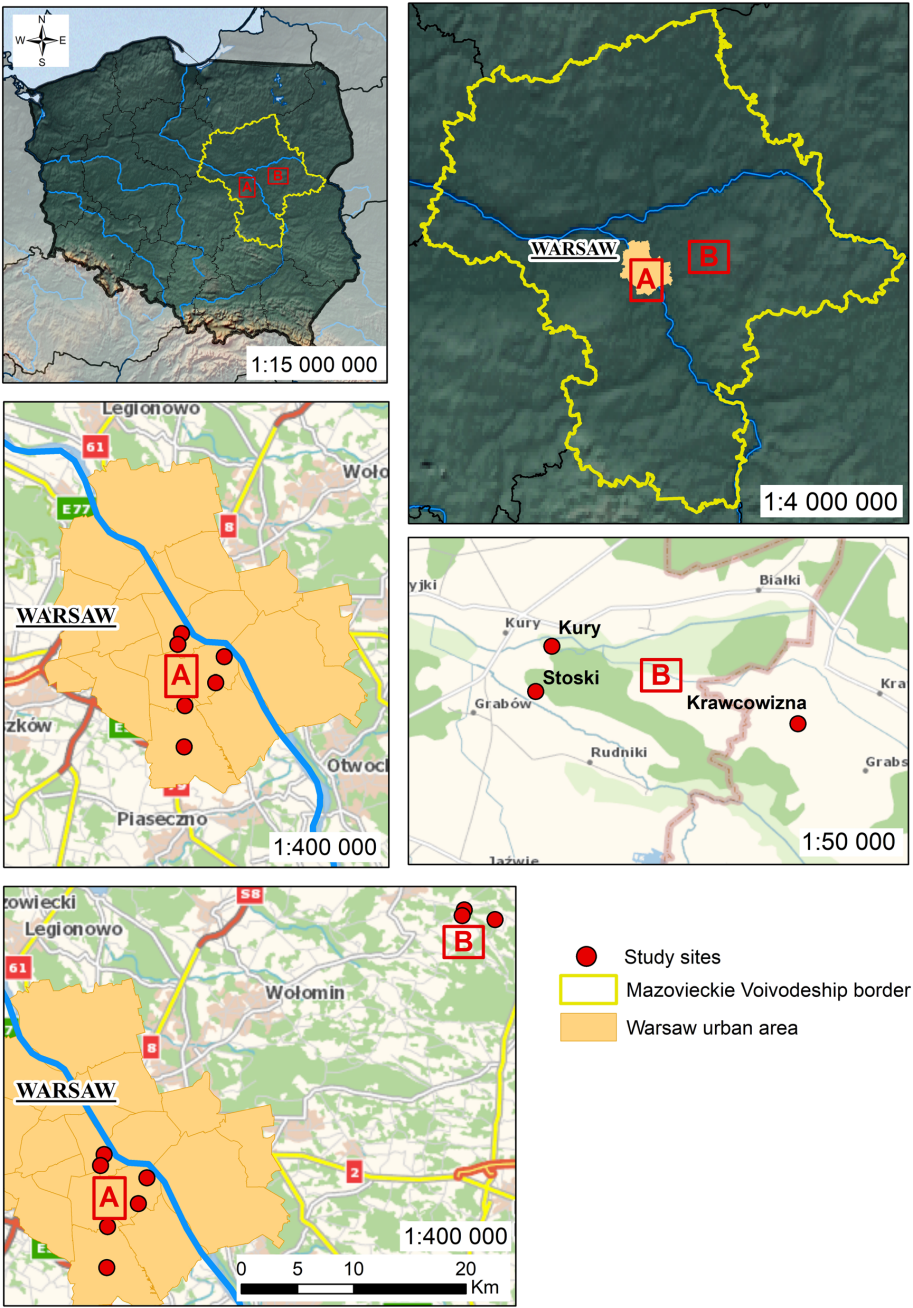
Maps of (**A**) urban (Warsaw) and (**B**) suburban study sites

The study was conducted in spring 2021. Systematic tick collection by dragging using a woolen blanket was performed every 1–2 weeks between mid-March and mid-June at 15 sites, encompassing 6 sites in Warsaw (urban areas) and 9 sites situated approximately 50 km northeast of Warsaw, in rural areas (near Stoski, Kury, and Krawcowizna villages) (suburban areas) (Fig. 1, Supplementary Files 1 and 2). Sites were selected to represent different types of habitats.

Three sites (one city forest [Kabacki forest] and two mixed forest sites outside Warsaw) were classified as forests (Table 1). In Kabacki forest, tick collections were focused in the children’s playground area “Leśne Przedszkole” (Forest Kindergarten), encompassing internal and external forested areas. Three sites in Warsaw (Warsaw Botanical Garden, Dolinka Służewiecka Park, Edward Rydz Śmigły Park) were classified as parks. Two abandoned grassy areas in Warsaw (grounds around Czerniakowskie lake and area in vicinity of Siekierkowski bridge) and two fallow lands in rural areas were grouped as fallow land. One site representing managed meadow in rural area was classified as meadow, in addition to two horse pastures (classified as pasture). Two sites in rural area represented crop fields (covered with winter/spring crops [cereals]) and one grassy area represented chicken enclosure, hosting about 30 hens and 2 geese (Table 1, Fig. 1 and Supplementary file 2). Detailed classification of habitats is provided in Table 1 and detailed description of sites is provided in Supplementary file 1.

**Table 1:** Tick density by site

	Warsaw (urban)				Suburban (Masovia)		
Area type and site	*D. reticulatus*	*I. ricinus*	Total ticks	Area type and site	*D. reticulatus*	*I. ricinus*	Total ticks
Forest				Forest			
Kabacki forest	0.01 ± 0.01	0.65 ± 0.25	0.67 ± 0.25	Forest 1. Stoski	1.50 ± 0.95	5.10 ± 2.34	6.60 ± 2.16
Total forest	0.01 ± 0.01	0.65 ± 0.25	0.67 ± 0.25	Forest 2. Krawcowizna	1.38 ± 1.05	3.38 ± 1.92	4.75 ± 1.55
				Total forest	1.44 ± 0.66	4.33 ± 1.49	5.78 ± 1.34
OPEN				OPEN			
Fallow land				Fallow land			
Siekierki fallow land	2.91 ± 0.37	0.35 ± 0.20	3.26 ± 0.36	Fallow land 1 Stoski	4.20 ± 2.62	2.40 ± 1.25	6.60 ± 2.66
Czerniakowskie lake fallow land	0.54 ± 0.12	0.05 ± 0.03	0.59 ± 0.12	Fallow land 2 Kury	0.70 ± 0.44	0.40 ± 0.19	1.10 ± 0.43
Total fallow land	1.10 ± 0.21	0.12 ± 0.06	1.22 ± 0.23	Total fallow land	2.45 ± 1.38	1.40 ± 0.68	3.85 ± 1.57
TOTAL OPEN	1.10 ± 0.21	0.12 ± 0.06	1.22 ± 0.23	Pasture			
Park				Pasture 1. Kury	0	0.20 ± 0.20	0.20 ± 0.20
Botanical garden	0	34.27 ± 6.81	34.27 ± 6.81	Pasture 2. Krawcowizna	0	0	0
Marshal Edward Rydz-Śmigły park	0	0	0	Total pasture	0	0.11 ± 0.11	0.11 ± 0.11
Służewiecka Valley park	0	0	0	Field			
Total park	0	12.2 ± 3.21	12.2 ± 3.21	Field 1. Stoski	0.30 ± 0.20	0.10 ± 0.10	0.40 ± 0.29
				Field 2. Krawcowizna	0.25 ± 0.25	0.88 ± 0.52	1.13 ± 0.43
				Total field	0.27±0.19	0.49±0.28	0.76±0.30
				Meadow			
				Meadow Stoski	1.40 ± 0.70	0	1.40 ± 0.70
				Chicken yard	0.20 ± 0.20	0	0.2 0 ± 0.20
				TOTAL OPEN	0.89 ± 0.40	0.50 ± 0.21	1.42 ± 0.48

Between 100 m and 500 m were dragged at every collection, and all collected ticks were fixed in 70% EtOH and transported to the laboratory of the Department of Eco-Epidemiology of Parasitic Diseases at the University of Warsaw. At every collection, temperature and humidity at ground level were measured using Humidity & Temperature Meter GM1361 (Benetech, Shenzhen, China). Ticks were always collected in the same areas and at the same time—at 10 AM, after the morning dew had evaporated. This timing was chosen to avoid high daytime temperatures and low humidity, which could reduce the collection of *Ixodes ricinus* ticks. Ticks were identified to species and stage level and counted, and tick densities were calculated per 100 m^2^ per one dragging event (each visit at specific site). Identification of collected ticks to species and developmental stage was done using morphological key by Estrada-Peña et al. (2018) [16] and a stereoscopic microscope, Zeiss Stemi 508.

The map (Fig. 1) presented in this paper was designed using the ArcGIS (ESRI) geoinformatic software version 10.8.2 (institutional license purchased by the University of Warsaw, Warsaw, Poland).

### Molecular detection of *Babesia*, *Borrelia*, and *Rickettsia* spp. in ticks from Warsaw

To verify the role of ticks from urban areas as vectors of key pathogens, a subset of ticks (*n* = 144, 72 adult *I. ricinus*—36 males, 36 females; and 72 *D. reticulatus*—36 males, 36 females) were examined for *Babesia*, *Borrelia*, and *Rickettsia* spp. Genomic DNA from ticks was isolated with a Genomic Tissue Spin-Up kit (A&A Biotechnology, Gdynia, Poland) from individual adults and stored at a temperature of −20 °C, according to manufacturer protocol. Genomic DNA was used for molecular screening for spirochaetes (*Borrelia burgdorferi* s.l.) by nested polymerase chain reaction (PCR) with the use of flagellin gene (*flaB*) marker, with genus-specific primers: 132f/905r and 220f/824r. The conditions for the PCR reaction have been described previously [11]. Molecular detection of *Babesia* spp. was performed by amplification of a 550 bp fragment of *18S* rDNA, as described previously [17]. Detection of *Rickettsia* spp. was made by using primers CS409 and Rp1258 which amplify a 769 bp fragment of the *gltA* gene. The conditions for the PCR reaction have been described previously [5]. PCR products were visualized on 1.5% agarose gels stained with Midori Green Stain (Nippon Genetics Europe, Düren, Germany).

### Statistical analysis

Tick densities were expressed and analyzed as arithmetic means for *I. ricinus* (all stages together) and *D. reticulatus* (questing adults) separately, and also for total ticks (*I. ricinus* + *D. reticulatus*). General linear models (GLM) (IBM Corporation, SPSS Statistics v. 29 software, Imago Pro v. 10) were used for the analysis of the mean tick ABUNDANCE (dependent variable) incorporating month (March, April, May, June) as a co-variate. Site of tick collection (1: forest; 2: park; 3: fallow land; 4: meadow; 5: pasture; 6: crop field; 7: chicken enclosure) or urban versus rural area or open versus forested habitat were used as fixed factors in appropriate GLMs.

Temperature at the ground level and humidity recorded at each collection event were tested against abundance of *I. ricinus, D. reticulatus*, and total ticks using Spearman rank correlation test (SPSS Statistics v. 25 software, Imago Pro v. 10). Spearman rank correlation test was also used to compare overall mean density of males versus females of each tick species by week.

## Results

In spring season 2021, 178 field collections of ticks were carried out, including 131 collections from urban sites and 47 collections from rural areas. Altogether, 738 ticks (385 adult *D. reticulatus* and 353 *I. ricinus*) were collected (Table 2).

**Table 2:** Species and stages of ticks collected during spring 2021

Tick species	Type of area	Number of ticks				Total
		Female	Male	Nymph	Larvae	
*Dermacentor reticulatus*	Urban (Warsaw)	125	99	0	0	224
	Suburban (rural)	118	43	0	0	161
*Ixodes ricinus*	Urban (Warsaw)	106	136	54	5	301
	Suburban (rural)	13	15	24	0	52
Total		362	293	78	5	738

**Fig. 2:**
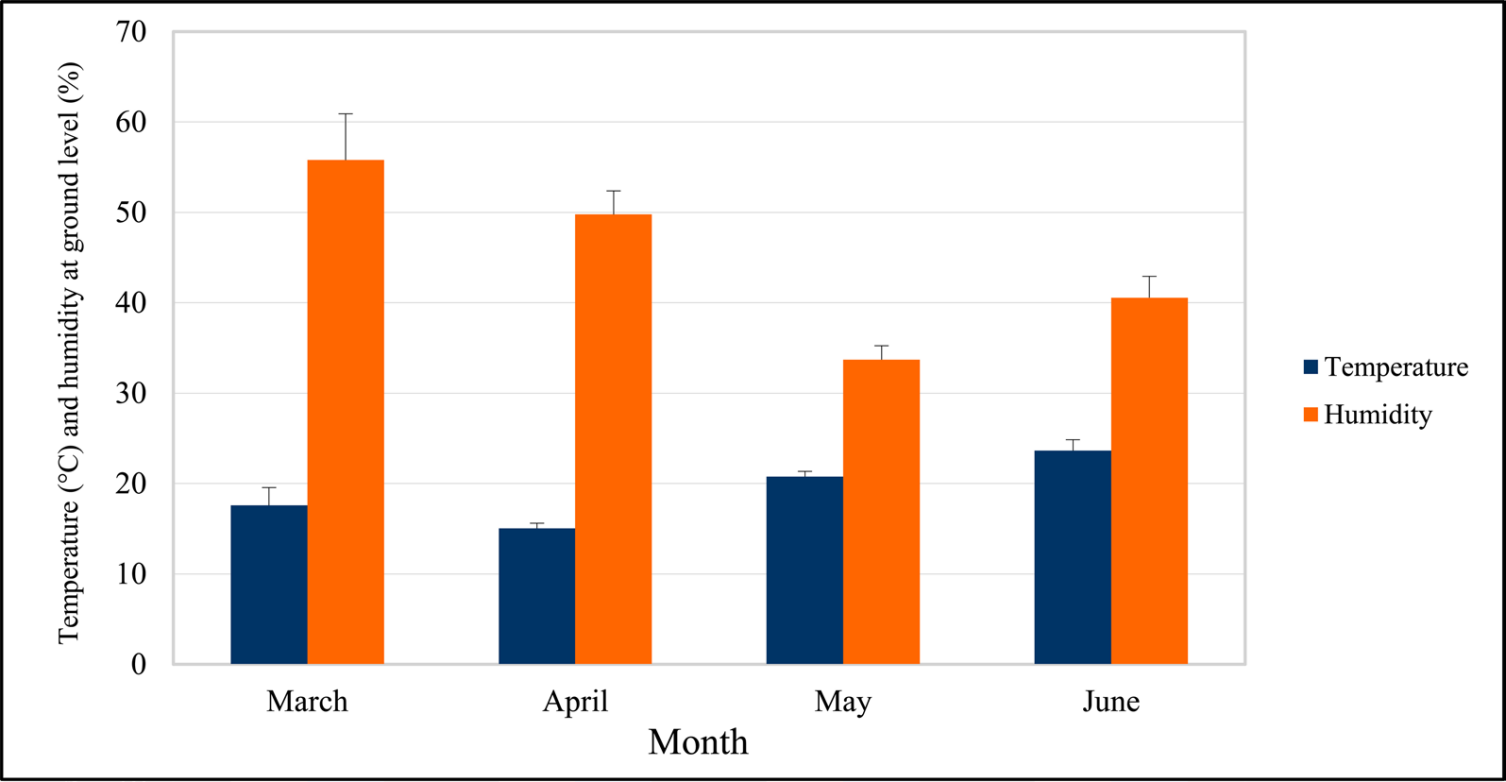
Variation in the total mean temperature and humidity at ground level during collections in spring season

There was a significant negative correlation (Spearman’s rho = −0.362, *P* < 0.001) between temperature and humidity measured at collection sites. As predicted, the mean air temperature at ground level was rising from March to June, while the mean humidity at ground level was slightly declining (Fig. 2).

### Factor affecting tick density

#### *Ixodes ricinus*

**Fig. 3:**
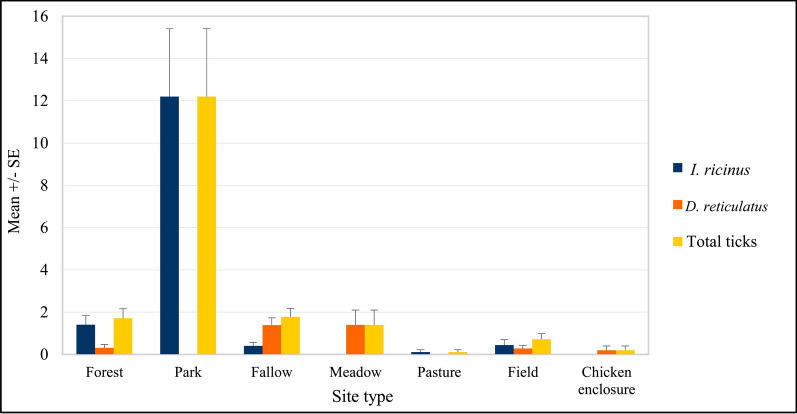
Overall mean density of tick species and total ticks by type of habitat

There was a highly significant effect of site type (main effect of site type on tick density: F_6, 177_ = 3.77*, P* < 0.001) on tick abundance (Fig. 3). The mean density of *I. ricinus* was highest in city parks, almost ten times higher than in forest sites (12.2 ± 3.21 versus 1.44 ± 0.66 ticks/100 m^2^; Table 1). However, this high mean density was affected by extremely high abundance of *I. ricinus* in the Botanical Garden (34.27 ± 6.81), while no ticks of both species were recorded in the other two parks (Table 1). Relatively high density was also observed in fallow lands and in crop fields. In the last case all ticks were collected at the field–forest border, where roe deer were regularly observed feeding on cereals. No ticks of this species were found in the chicken enclosure.

Although mean tick density was almost four times higher in urban than in rural areas (5.70 ± 17.5 versus 1.38 ± 2.67 ticks/100 m^2^), this difference was not quite significant (F_1, 177_ = 2.37, *P* = 0.13) due to great differences between urban sites as described above. Due to the same reason the difference in tick abundance between forested and open habitat was not quite significant (5.60 ± 17.4 versus 1.41 ± 2.77 ticks/ 100m^2^; F_1, 177_ = 2.58*, P* = 0.11).

**Fig. 4:**
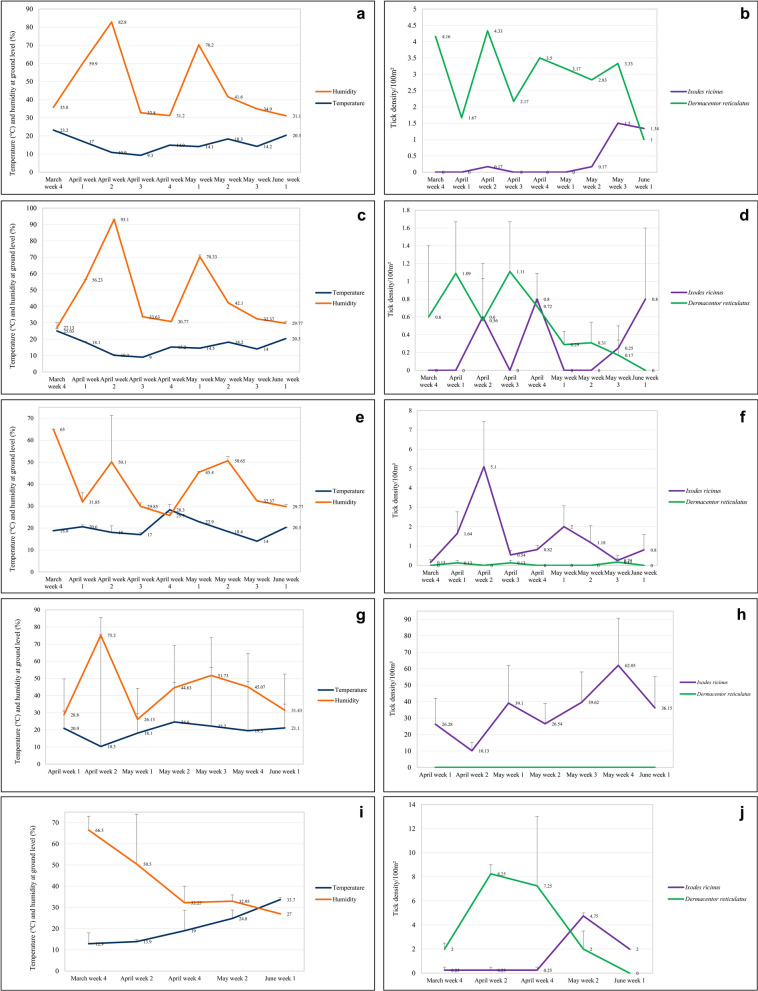

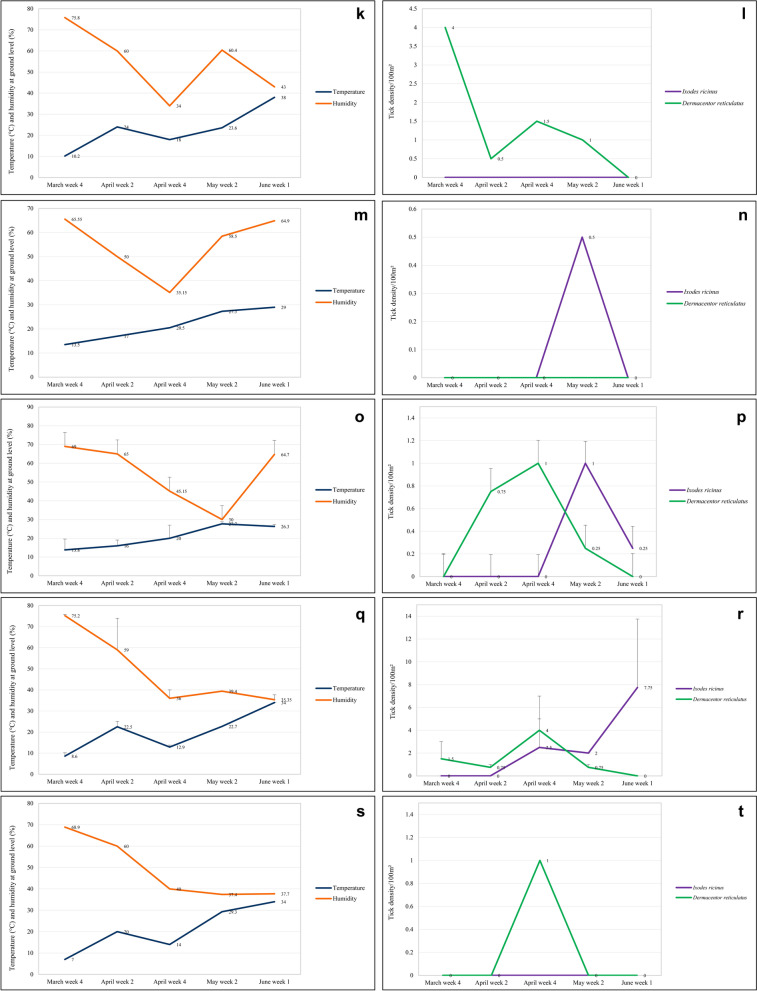
Spring changes in abiotic factors and tick abundance in positive urban sites and mean abundance in suburban habitats: **a**. temperature and humidity in Siekierki; **b**. tick density in Siekierki; **c**. temperature and humidity in Czerniakowskie lake; **d**. tick density in Czerniakowskie lake; **e**. temperature and humidity in Kabaty-Forest Kindergarten; **f**. tick density in Kabaty-Forest Kindergarten; **g**. temperature and humidity in Warsaw Botanical Garden; **h**. tick density in Warsaw Botanical Garden; **i**. temperature and humidity in the forest area; **j**. tick density in the forest area; **k**. temperature and humidity in the hay meadow area; **l**. tick density in the hay meadow area; **m**. temperature and humidity in the pasture area; **n**. tick density in the pasture area; **o**. temperature and humidity in the field area; **p**. tick density in the field area; **q**. temperature and humidity in the fallow land area; **r**. tick density in the fallow land area; **s**. temperature and humidity in chicken enclosure; **t**. tick density in chicken enclosure

There was no significant correlation between the abundance of *I. ricinus* and the temperature and humidity measured at collection sites (not significant, NS). However, a positive trend could be noted between the rise of temperature and tick density during spring season (Spearman’s rho = 0.152, *P* = 0.15) (Fig. 4a–t).

#### Spring dynamics

Changes in the mean density of *I. ricinus* in positive urban sites and in suburban habitat types with time, together with changes in the ground temperature and humidity, are presented in Fig. 4a–t. In general, the abundance of *I. ricinus* was the lowest in the beginning of spring season and was rising with collection weeks, peaking in May or April. Interestingly, ticks of this species were regularly collected from urban sites characterized by high human pressure/abundance, such as the Forest Kindergarten, Warsaw Botanical Garden, or vicinity of the Czerniakowskie lake.

**Fig. 5:**
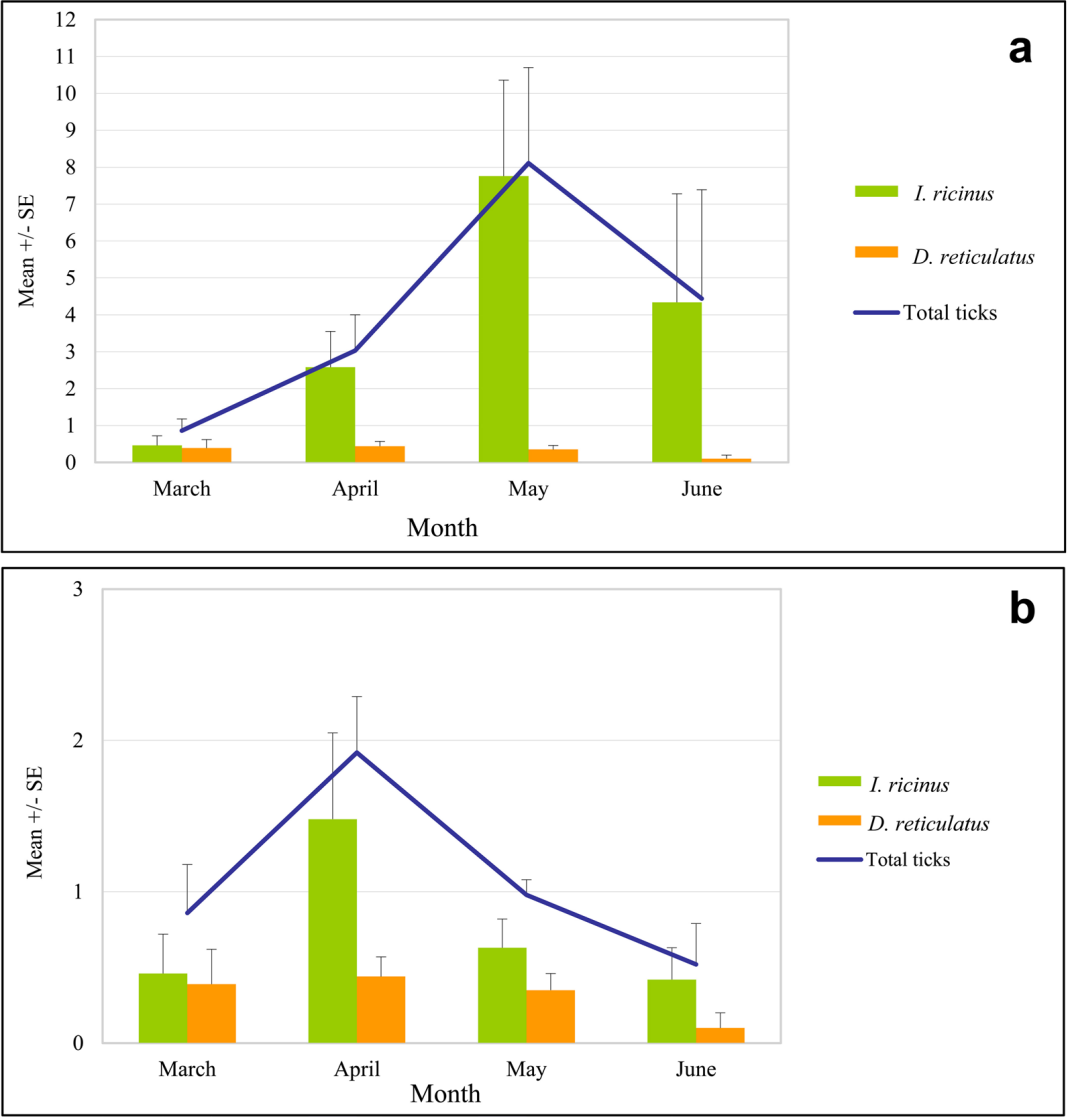
Spring changes in abundance of tick males and females in **a**. *Ixodes ricinus;* **b**. *Dermacentor reticulatus*

Spring dynamic in occurrence of males and females is presented in Fig. 5a. There was a strong positive correlation between density of males and females (Spearman rho’s = 0.960, *P* < 0.001), showing similar increase with time and both peaking in May.

### *Dermacentor reticulatus*

There was a highly significant effect of site type (main effect of site type on tick density: F_6, 177_ = 6.14*, P* < 0.001) on ornate dog tick abundance (Fig. 3). The highest mean abundance of *D. reticulatus* was observed in fallow lands, encompassing two urban and two rural areas (Table 1). This tick species was also collected in meadow, forests, and crop fields (Fig. 3). Similar to *I. ricinus*, ticks were collected at the field–forest border, where roe deer were regularly observed. Interestingly, no ticks of this species were collected in three city parks and in pastures but one female was collected from the chicken enclosure in April.

Mean tick density was almost three times higher in rural than in urban areas (0.87 ± 2.33 vs 0.31 ± 0.84 ticks/ 100m^2^) and this difference was significant (F_1, 177_ = 6.02*, P* = 0.015). However, the difference in tick abundance between forested and open habitats was not significant (Table 1, NS).

**Fig. 6:**
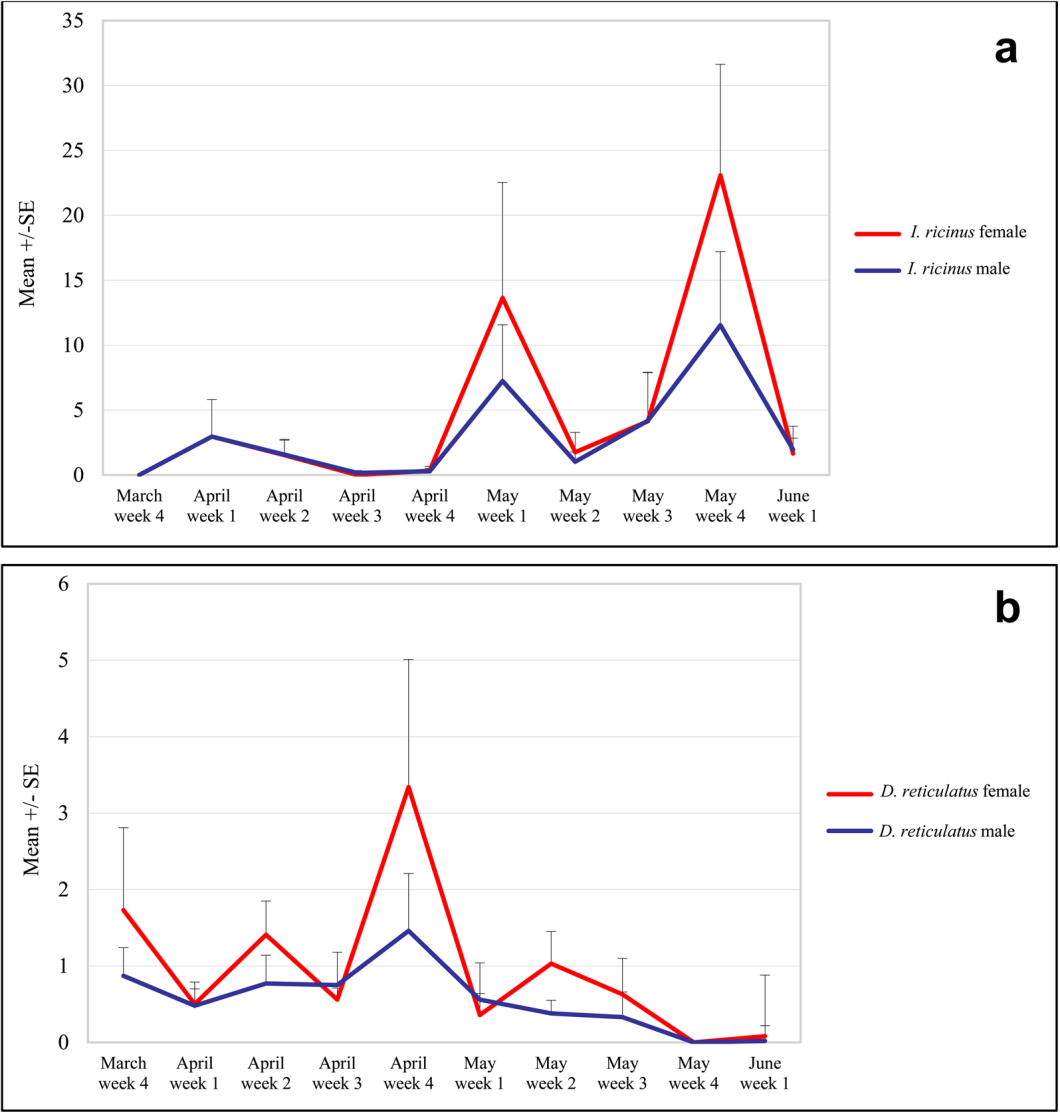
Overall mean density of tick species and total ticks in positive sites by month of the study: **a**. with all positive sites; **b**. without the Warsaw Botanical Garden

There was no significant correlation between the abundance of *D. reticulatus* and the temperature and humidity measured at collection sites (NS). However, there was almost a significant effect of the month co-variate on tick abundance (F_1, 177_ = 3.67*, P* = 0.06), with the highest tick density noted in April and lowest in June (Fig. 6a–b).

#### Spring dynamics

Changes in the mean density of *D. reticulatus* in positive urban sites and in suburban habitat types with time, together with changes in the ground temperature and humidity, are presented in Fig. 4a–t. Interestingly, ticks of this species started to be collected at the very beginning of the season, but the abundance of *D. reticulatus* was the lowest in the beginning and the end of the season and peaked mostly in April.

Spring dynamic in occurrence of males and females is presented in Fig. 6a–b. There was a positive correlation between density of males and females (Spearman’s rho = 0.806, *P* < 0.001), with some more differences in density of males and females than in *I. ricinus* at certain collection timepoints (Fig. 5b).

### Total ticks

Finally, we analyzed the effect of different factors on combined density of both tick species. Again, there was a significant effect of site type (main effect of site type on tick density: F_6, 177_ = 3.28*, P* = 0.004) on the abundance of total ticks (Fig. 3). The mean density of combined ticks was highest in city parks due to very high abundance of *I. ricinus* in the Warsaw Botanical Garden (Table 1). Relatively high tick density was also recorded in forests, fallow lands, and meadow. The lowest tick density was noted in chicken enclosure, crop fields, and pastures (Fig. 3).

The mean tick density was higher in urban than in rural areas (6.02 ± 17.5 versus 2.26 ± 3.59 ticks/100 m^2^), but this difference was not quite significant (F_1, 177_ = 1.73*, P* = 0.19) due to great differences between urban sites. The difference in tick abundance between forested and open habitat was close to statistical significance (mean tick density 6.11 ± 17.3 versus 1.71 ± 3.01 ticks/100 m^2^ in forested and open habitats, respectively; F_1, 177_ = 2.87*, P* = 0.092).

There was no significant correlation between the abundance of total ticks and the temperature and humidity measured at collection sites (NS).

#### Spring dynamics

Changes in the mean density of two species of ticks in positive urban sites and in suburban habitat types with time, together with changes in two abiotic factors, are presented in Fig. 4a–t. In general, the abundance of ticks was only slightly fluctuating through the spring because of the “exchange” between *D. reticulatus* and *I. ricinus* and was peaking in May (Fig. 6a), the month of high activity of both tick species; or in April, the month of the highest *D. reticulatus* activity if the exceptionally high numbers of *I. ricinus* from the Botanical Garden were removed from the dataset (Fig. 6b).

### Molecular detection of *Babesia*, *Rickettsia*, and *Borrelia* spp. in ticks from Warsaw

A total of 144 tick samples, comprising 72 *I. ricinus* (36 females, 36 males) and 72 *D. reticulatus* (36 females, 36 males), were screened for the presence of pathogens. DNA of *Borrelia burgdorferi* s.l. and/or *Borrelia miyamotoi* was detected in 14.3% of the *I. ricinus* samples, while it was not found in any of the *D. reticulatus* ticks. *Rickettsia* spp. was found in 64% of the *D. reticulatus* ticks and 19% of the *I. ricinus* ticks. No DNA of *Babesia* spp. was detected in any of the samples.

## Discussion

The main finding of our study is observation of different spring activity of two main tick species, with *D. reticulatus* ticks occurring much earlier than *I. ricinus*. Moreover, the spring activity of ticks seems more associated with temporal changes than the exact temperature of humidity levels. Habitat type was one of the most important factors affecting tick abundance. Interestingly, although the spring dynamic was similar for *I. ricinus* males and females, more disparity was observed between activity of *D. reticulatus* males and females.

We have observed marked differences in spring activity of two ticks species, both in urban and rural areas. In the beginning of spring season (mid-March), shortly after the rise of temperatures above zero, *D. reticulatus* ticks were able to be collected from a range of habitats. In the case of *I. ricinus*, the start of spring activity was recorded a few weeks later. This difference in tick activity was recorded before [18–20] and can be associated with habitat preferences: exposure to sun is higher in open habitats, such as fallow lands and meadows, and causes faster increase in soil temperature, resulting with termination of winter diapause of ornate dog ticks. Such increase in soil temperature is probably delayed in the case of shadowed forest habitats, causing delay in termination of *I. ricinus* winter diapause. Similarly, the greater exposure to the sun and rapid increase in the daily temperature in early June cause rapid cessation of *D. reticulatus* activity, while *I. ricinus* can be still active in more humid forest habitats. *Ixodes ricinus* is highly dependent on high humidity levels, which is why it primarily inhabits areas with dense vegetation cover, such as forests or shaded urban parks. In contrast, *D. reticulatus* is more tolerant of lower humidity and prefers open habitats, including grasslands, field edges, and fallow lands, as well as the outskirts of forests and parks [19, 20]. These ecological preferences likely explain the differences in the timing and patterns of spring activity observed in our study and should be considered in future research evaluating tick distribution in different environments.

Comparing our results with previous studies conducted in Poland, we observe similarities as well as differences in spring tick activity. In the study by Kiewra et al. (2014) [21] seasonal activity of *Ixodes ricinus* showed two peaks—spring and autumn, which was strongly correlated with temperature and humidity. Our results show that in the Warsaw area, *I. ricinus* begins activity in mid-April and peaks in May, which is consistent with previous studies conducted in Poland. In contrast to *I. ricinus,* the activity of *D. reticulatus* in our study was clearly earlier and began as early as March, reaching a maximum in April. These results differ from the study conducted in the Carpathian and Rzeszow regions, where *D. reticulatus* was found infrequently and only in single locations [22]. This may be due to differences in habitat preferences and microclimatic conditions between regions. A comparison with the study by Zając et al. (2021) [23], which focused on *I. ricinus* activity in eastern Poland, reveals further similarities and differences. Both studies confirm that *I. ricinus* reaches peak activity in May, supporting the general pattern of spring seasonality in Poland. However, while Zając et al. [23] observed this peak across the entire Lublin Province, our results indicate a more localized pattern in the Warsaw area, where tick activity was particularly high in urban green spaces, such as botanical gardens and parks. This suggests that *I. ricinus* is highly adaptable and can thrive not only in natural forested areas, as documented by Zając et al., but also in human-associated environments. Another important distinction is the role of microclimatic factors. Zając et al. [23] demonstrated that *I. ricinus* activity was significantly influenced by the saturation deficit, with higher temperatures and lower humidity reducing tick abundance. In contrast, our study did not find a significant correlation between tick density and specific weather parameters. Instead, the seasonal dynamics of *I. ricinus* in our study appeared to follow a more predictable temporal pattern, with a steady increase from April to May regardless of short-term fluctuations in temperature and humidity. This discrepancy may be due to differences in habitat structure—urban parks and botanical gardens may provide more stable microclimatic conditions, buffering against extreme variations in temperature and humidity, as opposed to the more exposed natural habitats analyzed by Zając et al. [23]. Overall, while both studies support the well-documented spring peak of *I. ricinus* activity in Poland, our findings emphasize the importance of urban environments as potential hotspots for tick populations.

Such differences in start of spring activity have consequence for the risk of contracting of TBD; the risk of contracting *B. canis* and canine babesiosis is high from early spring (March) until the end of May, while the risk of contracting *B. burgdorferi* s.l. and Lyme disease increases in late April and May, with increase in *I. ricinus* activity. Due to the “exchange” between *D. reticulatus* and *I. ricinus* from early to late spring, the risk of contracting any TBD is maintained high through the season. Both tick species are known as competent vectors for tick-borne encephalitis [14, 24, 25] and *Rickettsia* spp. [5, 14, 26, 27], thus the presence of both species at particular sites increases the risk of transmission to humans and animals.

Differences in activity of different tick species should be also considered in monitoring studies: lack of certain species at the time of sampling could result from delayed start of activity or earlier cessation of activity. Due to this, the Center for Disease Control and Prevention (CDC) recommends at least three sampling for ticks at certain sites during a season, to confirm presence/absence (https://www.cdc.gov/ticks/resources/TickSurveillance_Iscapularis-P.pdf).

We have also observed marked differences in occurrence of two tick species in different habitats. Both in rural and urban areas, there were habitats where ticks were not recorded (two urban parks) or present at very low density (pastures, chicken enclosure). All these habitats can be classified as well managed, with highly reduced vegetation cover, either by grazing or mowing. These two factors have been shown previously to reduce tick densities [28, 29]. Chickens are also likely to feed on ticks, exposed on grass blades while questing for hosts.

Another important factor that should be considered is the potential impact of tick control measures. The sites we selected for tick collection were actively visited by humans, domestic animals, and livestock, which means they could have been subjected to acaricide treatments or other protective measures. The use of chemical tick control in urban parks, recreational areas, or agricultural fields may influence tick abundance and activity patterns. However, this aspect was not the primary focus of our study and requires further investigation to determine its impact on tick population dynamics in urban and suburban environments [28, 29].

Tick densities were high in forests, fallow land, and crop fields visited by wildlife, as reported previously [29, 30], however the exceptionally high *I. ricinus* density was recorded in the urban site, the Warsaw Botanical Garden. This site seems well managed but vegetation cover has not undergone key changes since the foundation of the garden, providing a kind of perfect “refugium” for many arthropods, including ticks. This numerous population of common ticks in the Botanical Garden has been recorded for years [5, 11] despite the absence of large mammals in this small fenced area. High density of *I. ricinus* with confirmed presence of *B. burgdorferi* s.l. in ticks in this site constitute the real risk of contracting TBD for abundant visitors. Common ticks were also found continuously in Forest Kindergarten, in a fenced area extensively used on a daily basis as a playground and around this area. Currently, ticks and TBD constituted the greatest threat for children attending the kindergarten, with tick infestation in kids being reported 1–2 years by the parents. In our study, *Borrelia*-infected *I. ricinus* were also collected from that site. Because our study has demonstrated the effectiveness of tick (adults and nymphs) collection by dragging, this method was then used by parents and kindergarten staff on a weekly basis to control tick abundance in the area. It is also worth noting that spring tick activity is influenced not only by temperature and humidity, but also by other abiotic and biotic factors. Among them, the availability of hosts plays a crucial role, and this factor differs significantly between urban and suburban environments. In suburban and rural areas, large populations of deer and other wild mammals provide a continuous source of blood meals for both tick species, sustaining their populations throughout the year. In contrast, urban environments may offer fewer large hosts, leading to reliance on smaller mammals, birds, and even companion animals [31, 32]. These differences in host availability could explain variations in tick abundance and activity patterns between different habitat types.

In the current study, spring activity of ticks seemed more associated with temporal changes (exact season) than exact temperature of humidity levels. In other words, the dynamic of tick occurrence can be more the effect of evolution/fitness than of unpredictable exact abiotic factors. The lack of significant correlation between tick density and temperature/humidity could be also partially caused by our “selection” of weather conditions suitable for tick collection: collections could not be performed during rain or wet conditions. Such selection has certainly caused the bias toward warmer and dryer conditions/days.

Interestingly, although the spring dynamic was similar for *I. ricinus* males and females, more disparity was observed for *D. reticulatus* males and females. This can be a reason for the reported biases in proportion of females to males in some studies on *D. reticulatus* ticks [33].

## Conclusions

Due to the marked differences in spring dynamic of *D. reticulatus* and *I. ricinus*, the sampling effort should be repeated at least three times per season for accurate estimation of tick occurrence (presence/absence) and density, as recommended by the CDC. Due to “exchange” of tick species, total tick density remains high through the spring season of activity, which may result in high transmission of TBPs. Season (time of collection) may play a more important role for tick activity patterns than humidity or temperature fluctuation at certain sites. Tick densities are highly dependent on the habitat type and may be low in well-managed agricultural habitats (crop fields, pastures, chicken yard), but high in semi-natural (fallow lands, rural forests) habitats. Maybe surprisingly, numerous *I. ricinus* populations can be maintained in urban green areas (such as botanical gardens). Ticks from urban areas can serve as vectors of important TBPs (*B. burgdorferi* s.l., *Rickettsia* spp.).

## Supplementary Information

Additional file 1. Description of collection sites

Additional file 2. Spring changes in vegetation cover in chicken enclosure (fenced area) and fallow land (behind chicken enclosure) in Kury site by month: (a) 22 February; (b) 21 March; (c) 3 April; (d) 3 May; (e) 17 May 2021

## Data Availability

No datasets were generated or analyzed during the current study.

## Abbreviations

CDC: Center for Disease Control and Prevention
DNA: Deoxyribonucleic acid
EtOH: Ethyl alcohol
flaB: Flagellin
GLM: General linear models
gltA: Citrate synthase
NS: Not significant
PCR: Polymerase chain reaction
TBD: Tick-borne diseases
TBEV: Tick-borne encephalitis virus
TBP: Tick-borne pathogens
