# Supplementary material for: Are you ready for the tick season? Spring dynamic of tick diversity and density in urban and suburban areas
Source: Parasit Vectors. 2025 Apr 19;18:144. doi: 10.1186/s13071-025-06793-0 (PMC12009520; doi:10.1186/s13071-025-06793-0)
Supplement: Supplementary file 1 — Additional file 1. Description of collection sites [file 13071_2025_6793_MOESM1_ESM.docx]

**Collection sites**

Urban areas

**Siekierki (52°12'25.2"N; 21°05'11.0"E)**Located to the northeast of Warsaw, this urban site features an open area overgrown with tall, dry grass and scattered scrub trees. The area is inhabited by small mammals such as rodents and lizards. It is regularly frequented by individuals walking their dogs and is located near an outdoor shooting range, resulting in varied human and wildlife interactions.

**Czerniakowskie Lake (52°11'11.5"N; 21°04'27.4"E)**This natural water body covers approximately 19.5 hectares and is the largest of its kind in Warsaw. The lake measures about 1,780 meters in length and 100–180 meters in width. The southern shore is developed, with recreational areas like beaches and sports pitches, while the northern shore consists of undeveloped fallow land. This area supports diverse wildlife, including small and medium-sized mammals (e.g., European beavers, roe deer, wild boars, red foxes) and about 60 bird species. It is a popular destination for recreational activities, particularly on the southern shore.

**Marshall Edward Rydz-Śmigły Park (52°13'36.8"N; 21°01'53.6"E)**Situated centrally in Warsaw, this urban park spans approximately 90 hectares and is characterized by dense vegetation, including perennial trees, poplars, Turkish hazel, and tree sagebrush. The park hosts a variety of wildlife, such as birds, squirrels, and small rodents, and is a favored recreational area with cycle paths and rest spaces. Tick collections were conducted near park benches and tree canopies.

**Kabacki Forest (52°08'09.3"N; 21°01'51.1"E)**This managed forest covers 903 hectares and is located on the southern edge of Warsaw. Designated as a landscape reserve, it is ecologically significant and serves as a recreational hub for residents of Ursynów and Kabaty. Tick collections were carried out near a forest kindergarten on the forest’s edge.

**Botanical Garden of Warsaw University (52°13'04.7"N; 21°01'35.0"E)**Situated in central Warsaw, this botanical garden spans 5 hectares and contains approximately 5,000 plant species. It is also home to squirrels, small rodents, and stray cats. Tick collections were conducted along avenues and around grass beds.

**Służewiecka Valley Park (52°10'07.7"N; 21°02'00.5"E)**Located in the valley of the Służewiecka Stream, this park covers 22.8 hectares, featuring green areas and ponds dominated by willows and poplars. It is a habitat for birds, rodents, and squirrels, and is frequently visited by local residents. Tick collections were performed near the creek that flows through the park.

Suburban areas

**Sites Kury (52°41'52.7"N; 21°50'44.4"E)**

Rural area near village Kury. Tick collections were performed at horse pasture, grassy fallow land and in chicken enclosure. These free habitats were neighboring each other and covered an area of about 1 ha along the spring ‘Cienka’.

**Sites Stoski (52°40'72.2"N; 21°49'88.8"E)**

Rural area near villages Kury and Grabów. Tick collections took place in crop field (winter cereals) bordering birch forest, along the forest path in mixed forest (pines with oak trees), in managed meadow and in fallow land.

**Sites Krawcowizna (52°40'77.7"N; 21°56'55.5"E)**

ural area near village Krawcowizna. Ticks were collected in crop field (spring cereals) bordering pine forest; along the forest path in mixed forest (pines with acron trees) and in horse pasture.
