# Supplementary figures and images for: Are you ready for the tick season? Spring dynamic of tick diversity and density in urban and suburban areas
Source: Parasit Vectors. 2025 Apr 19;18:144. doi: 10.1186/s13071-025-06793-0 (PMC12009520; doi:10.1186/s13071-025-06793-0)

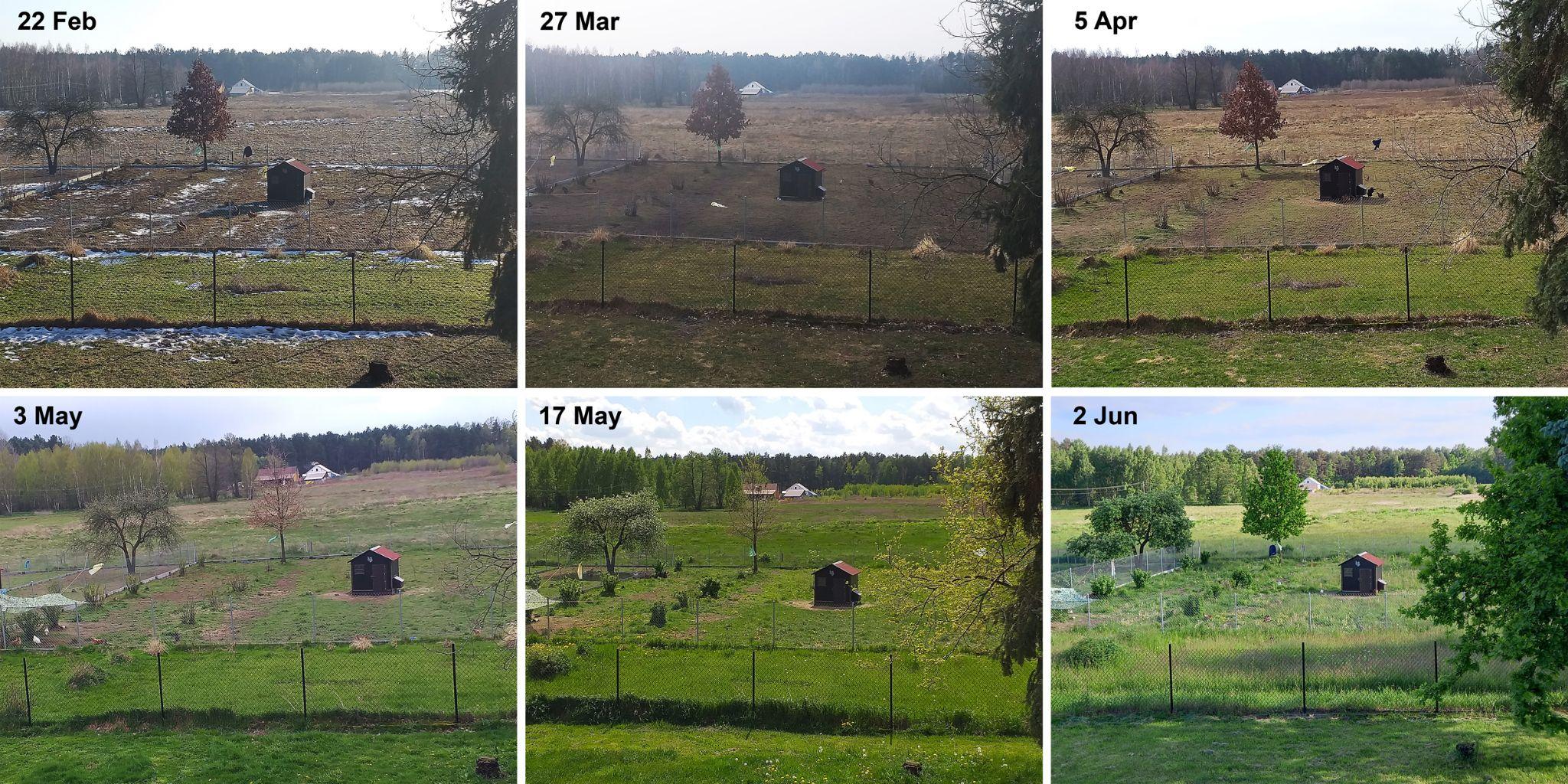

Supplement: Supplementary file 2 — Additional file 2. Spring changes in vegetation cover in chicken enclosure (fenced area) and fallow land (behind chicken enclosure) in Kury site by month: (a) 22 February; (b) 21 March; (c) 3 April; (d) 3 May; (e) 17 May 2021 [file 13071_2025_6793_MOESM2_ESM.docx]
